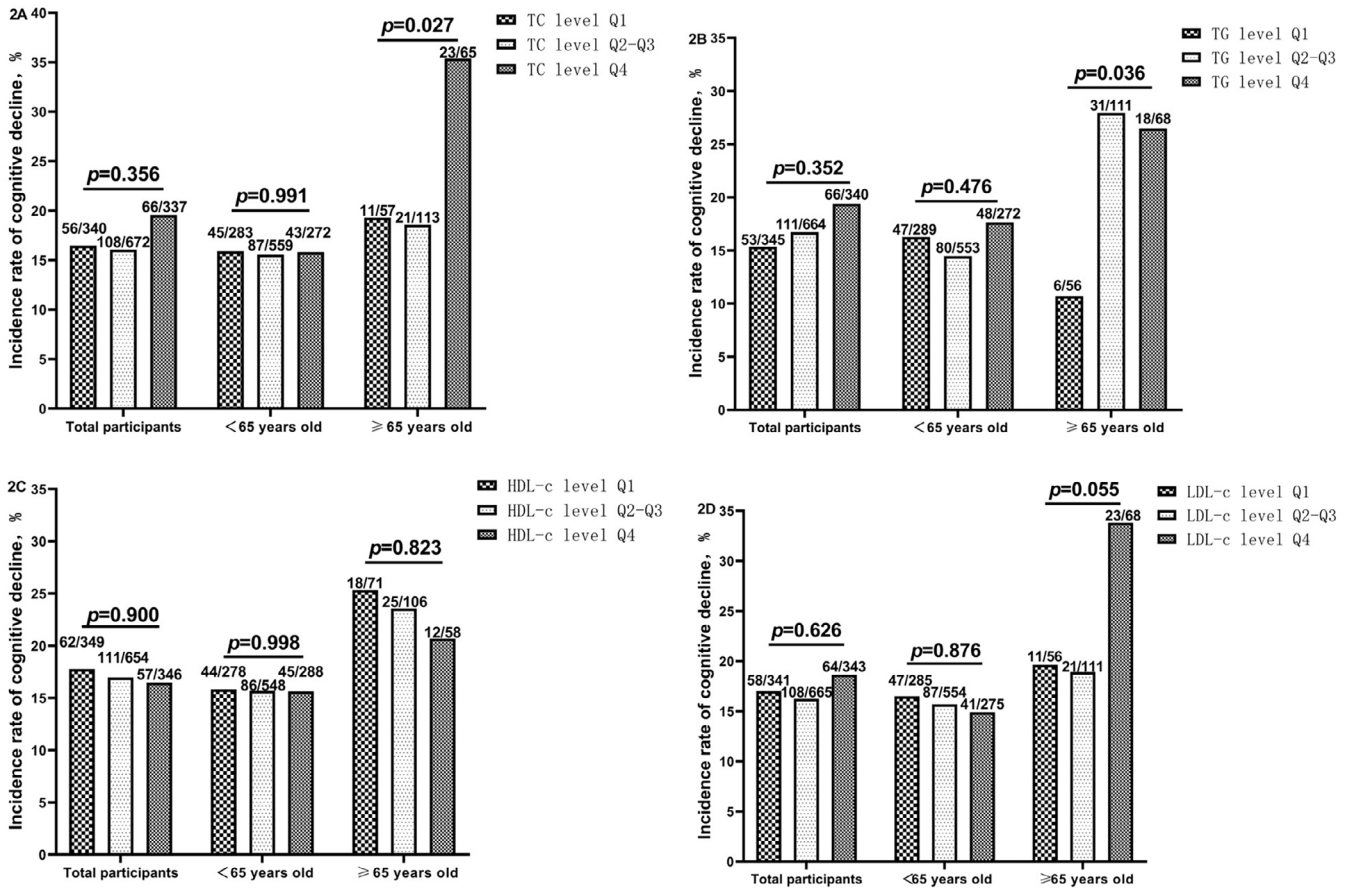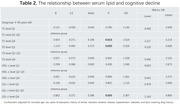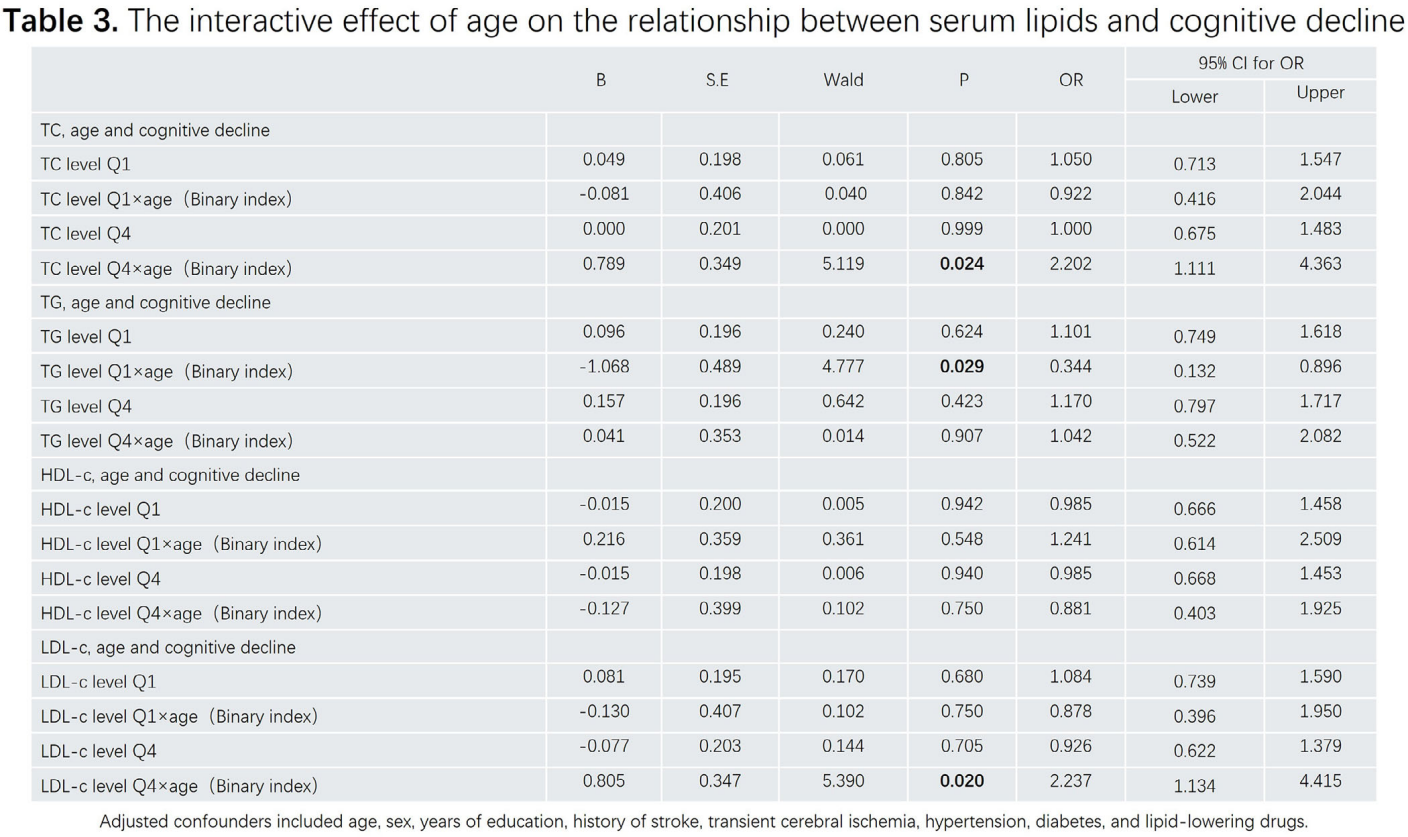# The relationship between baseline serum lipid levels and cognitive decline: A community‐based 4‐year prospective cohort study

**DOI:** 10.1002/alz70856_101410

**Published:** 2025-12-25

**Authors:** Ningwei Hu, Shan Wei, Liangjun Dang, Chen Chen, Ling Gao, Jingyi Wang, Jin Wang, Qiumin Qu, Suhang Shang

**Affiliations:** ^1^ The First Affiliated Hospital of Xi'an Jiaotong University, Xi'an, Shaanxi, China; ^2^ Huxian Hospital of Traditional Chinese Medicine, Xi'an, Shaanxi, China

## Abstract

**Background:**

Dyslipidemia as a risk factor for cognitive impairments has been known widely, however the effects of serum lipids levels on cognitive decline (CD) had not been determined.

**Method:**

This cohort initiated in 2014, and two follow‐up visits were conducted in 2016 and 2018. The Mini‐Mental State Examination (MMSE) was used to assess cognitive function and a drop of ≥2 points in MMSE score was defined as CD in 4‐years follow‐up. Baseline lipid levels [total cholesterol (TC), triglyceride (TG), high‐density lipoprotein cholesterol (HDL‐c), low‐density lipoprotein cholesterol (low‐density lipoprotein cholesterol (LDL⁃c)] were converted into 3 classifications based on 25% and 75% quantile [level Q1 (≤25%), level Q2‐Q3 (25%‐75%) (reference group), level Q4 (≥75%)]. The relationship between serum lipid and CD was analyzed by multivariate logistic regression. Interaction effect (IE) and subgroup analysis (< 65 years vs ≥65 years) has also been used.

**Result:**

There were 1,349 participants in the analysis, with 230 cases (17.05%) of CD. In the subgroup < 65 years, incidence rate has no differences among the 3 classifications within each lipid index. No associations between serum lipid and CD were found. In the subgroup ≥65 years, incidence rate of CD was increased in TC and LDL‐c level Q4 and was decreased in TG level Q1 (Figure 1). Compared with reference group, TC level Q4 (≥5.61 mmol/L) was associated with an increased risk of CD (OR =2.519, 95%CI 1.217‐5.214, *P* = 0.013) (Tab 1). Age had an IE (OR_TC level Q4× age_ = 2.202, 95%CI 1.111‐4.363, *P* = 0.024) (Tab 2); TG level Q1 (≤ 1.03 mmol /L) was associated with a lower risk of CD (OR = 0.318, 95%CI 0.120‐0.838, *P* = 0.020). Age had an IE (OR_TG level Q1× age_ = 0.344, 95%CI 0.132‐0.896, *P* = 0.029); LDL‐c level Q4 (≥3.81 mmol/L) was associated with an increased risk of CD (OR = 2.367, 95%CI 1.143‐4.900, *P* = 0.020). Age had an IE (OR_LDL‐c level Q4× age_=2.237, 95%CI 1.134‐4.415, *P* = 0.020).

**Conclusion:**

In participants over 65 years old, high baseline levels of TC and LDL‐c, low baseline levels of TG was associated with CD. These suggested that dyslipidemia patients may accelerate CD in older people.